# 

**Published:** 1883-04

**Authors:** 


					﻿APRIL, 1883.
/thirtieth year.
HALL'S
.JWBSÀ1
OF
HEALTH
Guaranteed Circulation 200,000 Copies in 12 Months.
E. H. GIBBS, A.M., M.D., Editor,
No. 21 CLINTON PLACE, EIGHTH STREET, NEW TOBK.
TERMS: $1 a Year.
Single unrulier, 10 ctsX
' $
				

